# Predictors and a prediction model for positive fine needle aspiration biopsy in C-TIRADS 4 thyroid nodules

**DOI:** 10.3389/fendo.2023.1154984

**Published:** 2023-07-24

**Authors:** Zhijie Yang, Xin Gao, Lu Yang

**Affiliations:** Department of Breast and Thyroid Surgery, Second Affiliated Hospital of Chongqing Medical University, Chongqing, China

**Keywords:** thyroid nodules, C-TIRADS, fine needle aspiration biopsy, predictor, prediction model 444, 95% Cl=0.296~0.666, low echo (OR=3.549)

## Abstract

**Objectives:**

To screen out the predictors and establish a prediction model of positive fine needle aspiration biopsy (FNAB) in the Chinese Guidelines for Malignant Risk Stratification of Thyroid Nodule Ultrasound (C-TIRADS) 4 thyroid nodules, and this nomogram can help clinicians evaluate the risk of positive FNAB and determine if FNAB is necessary.

**Methods:**

We retrospectively analyzed data from 547 patients who had C-TIRADS 4 thyroid nodules and underwent fine-needle aspiration biopsy (FNAB) at the Second Affiliated Hospital of Chongqing Medical University between November 30, 2021 and September 5, 2022. Patients who met our inclusion criteria were divided into two groups based on positive or negative FNAB results. We compared their ultrasound (US) features, BRAF V600E status, thyroid function, and other general characteristics using univariate and multivariate logistic regression analyses to identify independent predictors. These predictors were then used to construct a nomogram. The calibration plot, area under the curve (AUC), and decision curve analysis were employed to evaluate the calibration, discrimination, and clinical utility of the prediction model.

**Results:**

Out of 547 patients, 39.3% (215/547) had a positive result on fine-needle aspiration biopsy (FNAB), while 60.7% (332/547) had a negative result. Univariate logistic regression analysis revealed no significant differences in TPOAb, TgAb, TSH, Tg, nodule location, sex, or solid status between the two groups (P>0.05). However, age, nodule size, internal or surrounding blood flow signal, microcalcifications, aspect ratio, morphology, and low echo showed significant differences (P<0.05). Multivariate logistic regression analysis was conducted to explore the correlation between potential independent predictors. The results showed that only age (OR=0.444, 95% Cl=0.296~0.666, P<0.001), low echo (OR=3.549, 95% Cl=2.319~5.432, P<0.001), microcalcifications (OR=2.531, 95% Cl=1.661~3.856, P<0.001), aspect ratio (OR=3.032, 95% Cl=1.819~5.052, P<0.001), and morphology (OR=2.437, 95% Cl=1.586~3.745, P<0.001) were independent predictors for a positive FNAB. These variables were used to construct a prediction nomogram. An ROC curve analysis was performed to assess the accuracy of the nomogram, and AUC=0.793, which indicated good discrimination and decision curve analysis demonstrated clinical significance within a threshold range of 14% to 91%.

**Conclusion:**

In conclusion, 5 independent predictors of positive FNAB, including age (≤45 years old), low echo (yes), microcalcifications (yes), aspect ratio (>1) and morphology (irregular), were identified. A nomogram was established based on the above 5 predictors, and the nomogram can be used as a complementary basis to help clinicians make decisions on FNAB of C-TI-RADS 4 thyroid nodules.

## Introduction

The rapid increase in morbidity due to thyroid nodules is possibly due to the widespread application of modern imaging modalities, such as ultrasound (US) and computed tomography (CT) (1). About 60% of adults have at least 1 thyroid nodules, but only approximately 5% are confirmed as malignant tumors (2). It seems that overdiagnosis and overtreatment are inevitable, which are linked to potentially unnecessary costs and nonnegligible iatrogenic injury for patients (2, 3). Hence, it is essential to accurately diagnose and treat thyroid nodules. Fine needle aspiration biopsy (FNAB) is an effective way to identify thyroid nodules, especially when the added BRAF V600E test increases the accuracy of FNAB.

The indications for FNAB are often based on thyroid ultrasound, and thyroid image report and data system (TI-RADS) 4 thyroid nodules are often the targets of FNAB in clinical practice. TI-RADS 4 thyroid nodules can be divided into 3 subtypes (4A, 4B and 4C) based on various vital ultrasound features (4), and these types roughly represent the risk of thyroid carcinoma from 2% to 90%. Without further accurate information, FNAB is often recommended for patients to avoid missed diagnosis. Several versions of the TIRADS are used in the clinic, including the Korean Thyroid Association guideline (K-TIRADS), the American Thyroid Association guideline (ATA guideline), the American College of Radiology guideline (ACR-TIRADS), and the Chinese guideline (C-TIRADS), and different versions are slightly different. In the detection of thyroid carcinoma, C-TIRADS exhibited superior diagnostic performance and relatively lower rates of unnecessary biopsy, as demonstrated by a study (5). Additionally, ultrasound doctors usually give reports relying on the C-TIRADS in China. The ultrasound features that may relate to raising concern for thyroid malignancy include low echo, solid, microcalcifications, aspect ratio, internal or surrounding blood flow signal, and morphology (regular or irregular) (6). A study showed that 3 ultrasound features of solid thyroid nodules, including microcalcifications, marked low echo, and aspect ratio >1, are valuable in the diagnosis of thyroid malignancy (7). Another study indicated that the proportion of malignancies was 16.0% in 4A, 43.2% in 4B and 72.7% in 4C (8). In fact, quite a few TI-RADS 4 thyroid nodules are benign, and FNAB is unnecessary for them. Moreover, ultrasound greatly depends on the the experience of the inspectors and quality of equipment (3, 9, 10). Therefore, it is controversial to solely rely on TI-RADS to make the decision of FNAB.

Age is a vital factor in thyroid carcinoma staging, and age >45 years indicates an advanced stage. However, a study showed that among the patients who received FNAB and were finally confirmed as having a malignant nodule, the number of patients aged ≤45 years old was twice as high as that of patients aged >45 years old. Another study showed that the risk of thyroid nodules increased with age, while the possibility of malignancy decreased (11). Moreover, sex is significantly linked to the morbidity of thyroid carcinoma, and females have a higher morbidity. Nevertheless, a study confirmed that male patients who received FNAB may be more likely to obtain malignant or determinate results (12). In most studies, the authors believe that the size of thyroid nodules is uncorrelated with their oncologic properties (13), but a study showed that a nodule with size of 1-1.9 cm was more likely to be benign (14). Thyroid function is also a group of important potential predictors. Anti-thyroperoxidase antibody (TPOAb) and thyroglobulin antibody (TgAb) are significant predictors in many studies, and free thyroxine (T4), triiodothyronine (T3), thyroglobulin (Tg), and thyroid-stimulating hormone (TSH) are controversial (15–20). In addition, a study has demonstrated that the location of thyroid nodules, particularly in the isthmus region, is a significant independent predictor for malignancy (21). Hence, more potential predictors, such as age, sex, nodule size, thyroid function, and nodule location, need to be included to predict malignancy.

As mentioned, ultrasound-guided FNAB is more important in the diagnosis of thyroid nodules (22). The majority of complications following a fine-needle biopsy, such as pain and small hematomas, have a low occurrence rate and typically resolve on their own. Severe complications, including significant hematomas, infections, and tumor dissemination, are exceedingly rare (23). Moreover, the patients may have dizziness, palpitation, shortness of breath, hypotension and other discomfort during FNAB. Accordingly, FNAB should be cautiously recommended. Even for C-TIRADS 4 thyroid nodules, there are still many negative results after FNAB. To avoid or reduce the waste of medical resources and invasive operations, it is crucial to predict FNAB results in C-TIRADS 4 thyroid nodules.

Nomograms, as a common prediction model, have widespread use for physicians’ clinical decisions (24). It has many advantages, including high accuracy, simple calculation, and easy understanding. The objective of this study was to identify significant predictors among multiple medical information sources and construct a prediction model (nomogram) for positive fine-needle aspiration biopsy results.

## Materials and methods

### Patients

All patients were examined with color Doppler ultrasound and underwent ultrasound-guided FNAB at the Second Affiliated Hospital of Chongqing Medical University (Chongqing, China). The inclusion criteria were as follows: (1) patients whose ultrasound reports indicated C-TIRADS 4 thyroid nodules; (2) the patient had never been diagnosed with thyroid malignancy; (3) the maximum diameter of the thyroid nodule was more than or equal to 5 mm; (4) cytological examination and BRAF V600E mutation analysis were performed on the FNAB specimen; and (5) the patient had complete medical information. The exclusion criteria were as follows: (1) history of thyroid malignancy; (2) history of other malignancies, including breast cancer and lung cancer; (3) history of thyroid surgery or iodine-131 treatment; (4) maximum diameter of the thyroid nodule less than 5 mm; and (5) incomplete medical information. 547 patients were included in this study between December 2021 and September 2022 based on the inclusion and exclusion criteria. In this study, positive FNAB results met the following criteria: positive cytology results including all kinds of malignant tumors and suspicious malignant tumors with or without mutational BRAF V600E genes. In contrast, negative FNAB results met the following criteria: negative cytology results including all kinds of benign cells, such as inflammatory cells and follicular epithelial cells.

### Ultrasonography

Before FNAB, thyroid nodules were examined using color Doppler ultrasound (Philips IU22, L12-5 linear array probe, 5-12 MHz, Philips Medical). Reports showed internal or surrounding blood flow signal (poor/rich), solid (yes/no), nodule size (maximum diameter ≥1.0 cm/<1.0 cm), microcalcifications (yes/no), aspect ratio (≤1/>1), morphology (regular/irregular), low echo (yes/no).

### C-TIRADS

The expert committee established the C-TIRADS by counting the score of suspicious ultrasound features (solid, microcalcification, low echo, blurry borderline, irregularity boundary, extrathyroid invasion, and vertical position are suspicious malignant ultrasound features, while the comet tail sign is a benign feature). Each suspicious malignant sign is 1 point, while the comet tail sign is -1 point. The final score of the thyroid nodules is calculated by adding the scores of all individual features. A score of 1 corresponds to C-TIRADS 4A, a score of 2 corresponds to C-TIRADS 4B, and a score of 3-4 corresponds to C-TIRADS 4C.

### US-Guide FNAB

**Figure 1:**
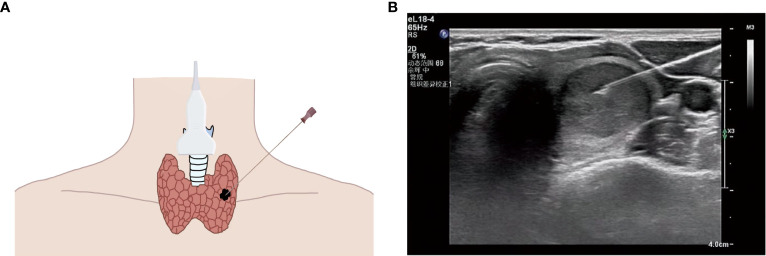
**(A)** Schematic diagram of ultrasound (US)-guided fine needle aspiration biopsy (thyroid nodule). **(B)** US image of fine needle aspiration (thyroid nodule).

The patients were placed in the supine position. Before the procedure, a brief preliminary ultrasound of the thyroid was performed to determine the location of the thyroid nodule. The skin was marked at the puncture site according to the determined nodule position, disinfected with iodophor and covered with a sterile surgical towel conventionally. Similarly, the ultrasound probe was disinfected with an iodophor, and the iodophor could also be used as a coupling agent. Lidocaine (2%, 1 ml) was subcutaneously injected for local anesthesia. The probe was placed over the target nodule in the transverse position. A fine needle (GALLINI S. R. L, Italy) was used to puncture the thyroid nodule from the marked site along the long axis of the probe under the guidance of ultrasound (**Figure 1**). After removing the core, the fine needle was repeatedly extracted and inserted into the nodule several times and then removed quickly. One part of the specimen was fixed onto a designated slide and delivered to the Department of Pathology of the Second Affiliated Hospital of Chongqing Medical University (Chongqing, China) for cytological examination; the other part of the specimen was placed into 1 ml of saline and delivered to the Molecular Diagnostic Laboratory of the Second Affiliated Hospital of Chongqing Medical University (Chongqing, China) for BRAF V600E mutation analysis.

### BRAF V600E mutation analysis

The reaction mixture was thawed at room temperature and agitated with a vortex for 15 seconds. It was then centrifuged at 2,000 × g for 15 seconds at room temperature. The reaction mixture (35 µl) was combined with 0.4 µl of Taq enzyme and then added to a PCR tube, which was kept in an ice bath. 5 µl of DNA sample (2-5 ng), 5 µl of positive control, and 5 µl of negative control were added to each PCR tube separately. The PCR tubes were spun at 2,000 × g for 1 minute at room temperature and subjected to thermocycling using a CFX96 thermocycler (Bio-Rad Laboratories, Inc.). The following thermocycling protocol was used for quantitative PCR: an initial denaturation step at 95°C for 5 minutes; 15 cycles of 95°C for 25 seconds, 64°C for 20 seconds, and 72°C for 20 seconds; and an additional 31 cycles of 93°C for 25 seconds, 60°C for 35 seconds, and 72°C for 20 seconds. The carboxyfluorescein (FAM) and hexachlorofluorescein (HEX) signals were detected at 60°C during the final set of cycling conditions. The relative amount of DNA was quantified using the 2−DDCq method (25). If the sample’s FAM signal Cq value was ≥28, it was considered negative for the BRAF V600E mutation. If the sample’s FAM signal Cq value was <28, it was considered positive for the BRAF V600E mutation, as per the manufacturer’s instructions (26, 27).

### Statistical analysis

Statistical analyses were conducted using SPSS version 26.0. Univariate and multivariate logistic regression analyses were performed to screen out the independent predictors of positive FNAB. The prediction nomogram based on independent predictors was developed by using R software (Version 4.2.1). A calibration curve was plotted to evaluate the calibration of the nomogram. Discrimination ability was measured by area under the curve (AUC) in the training cohort and validated through two internal validation cohorts, which were randomly divided from the training cohort: validation cohort 1 (n=274) and validation cohort 2 (n=273). Furthermore, a decision curve analysis was employed to determine the clinical utility of the nomogram by measuring the net benefit at the different thresholds.

## Results

### Patient characteristics

**Figure 2:**
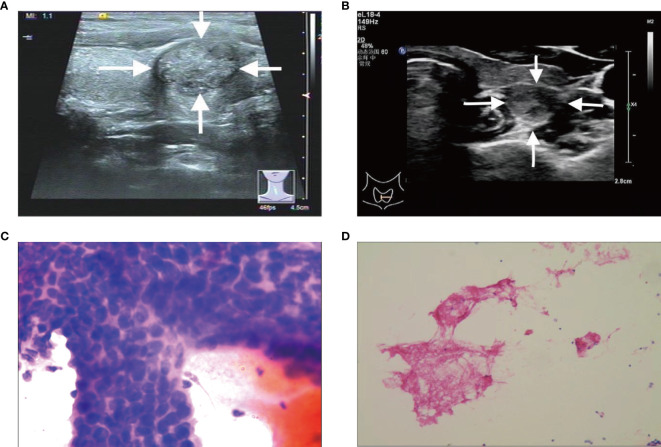
**(A, C)** A C-TIRADS 4A thyroid nodules patient (31-year-old male) with positive fine needle aspiration biopsy (FNAB). **(A)** The thyroid nodule had microcalcifications on US images. **(C)** Cytological examination indicated suspicious malignant tumor. **(B, D)** A C-TIRADS 4A thyroid nodules patient (40-year-old female) with negative FNAB. **(B)** The thyroid nodule had irregular morphology on US images. **(D)** Cytological examination indicated follicular epithelial cells.

**Figure 3:**
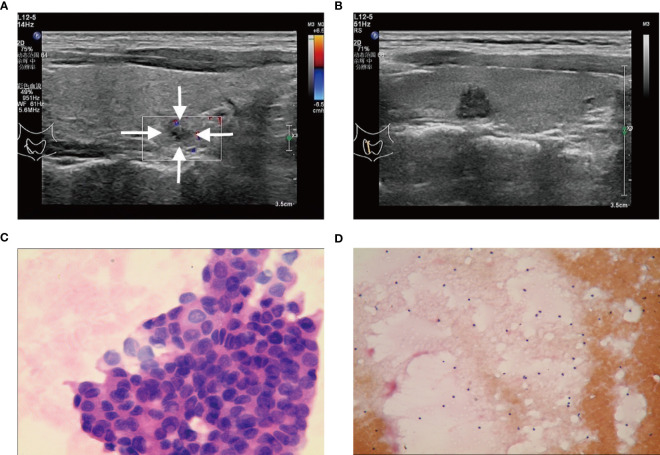
**(A, C)** A C-TIRADS 4B thyroid nodules patient (25-year-old female) with positive fine needle aspiration biopsy (FNAB). **(A)** The thyroid nodule had a low echo and irregular morphology on US images. **(C)** Cytological examination indicated suspicious malignant tumor. **(B, D)** A C-TIRADS 4B thyroid nodules patient (36-year-old male) with negative FNAB. **(B)** The thyroid nodule had a low echo and irregular morphology on US images. **(D)** Cytological examination indicated inflammatory.

**Figure 4:**
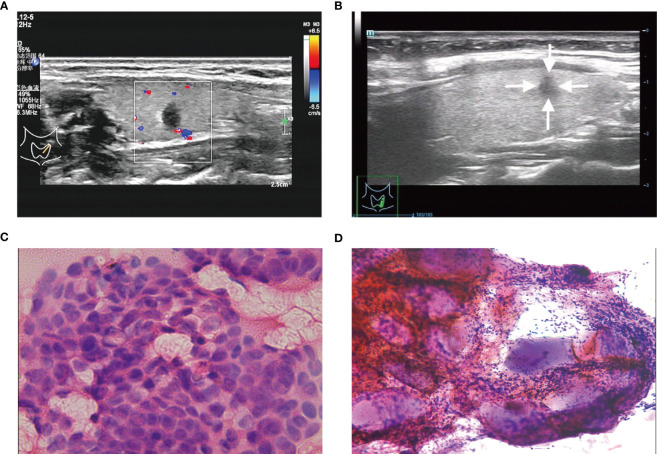
**(A, C)** A C-TIRADS 4C thyroid nodules patient (32-year-old female) with positive fine needle aspiration biopsy (FNAB). **(A)** The thyroid nodule had a low echo, aspect ratio >1 and irregular morphology on US images. **(C)** Cytological examination indicated suspicious malignant tumor. **(B, D)** A C-TIRADS 4C thyroid nodules patient (28-year-old female) with negative FNAB. **(B)** The thyroid nodule had a low echo, aspect ratio >1 and irregular morphology on US images. **(D)** Cytological examination indicated inflammatory cell, follicular epithelial cells and gelatine.

A total of 547 patients were finally confirmed to have C-TIRADS 4 thyroid nodules and underwent FNAB, among which 438 (80.07%) were women, and 109 (19.93%) were men. Of the 547 patients, 226 (41.31%) were ≤45 years old, and 321 (58.69%) were >45 years old (**Table 1**). After cytological examination and BRAF V600E mutation analysis, positive FNAB was confirmed. Of all patients, 215 (39.31%) had a positive FNAB (**Figures 2**, **3**, **4A, C**), while 332 (60.69%) did not (**Figures 2**, **3**, **4B, D**). Among the positive FANB, there were 125 patients were confirmed as thyroid cancer after surgery, only 9 patients were confirmed as benign tumor, and 81 patients selected follow-up. It can be roughly concluded that the accuracy of FNAB for C-TIRADS 4 thyroid nodules in our center was 93.28% (125/134), and the follow-up rate of positive FNAB was 37.67% (81/215).

**Table 1:** Univariate analysis of the relationship between medical information and positive FNAB.

Medical information	Positive FNAB		χ^2^	p
	yes (n=215)	no (n=332)		
Age (year)			26.893	<0.001
≤45	118 (52.21)	108 (47.79)		
>45	97 (30.22)	224 (69.78)		
Gender			0.223	0.636
female	170 (38.81)	268 (61.19)		
male	45 (41.28)	64 (58.72)		
Nodule size (cm)			6.742	0.009
<1.0	135 (44.12)	171 (55.88)		
≥1.0	80 (33.20)	161 (66.80)		
Internal or surrounding blood flow signal			4.908	0.027
no	81 (46.02)	95 (53.98)		
yes	134 (36.12)	237 (63.88)		
Tg			2.166	0.339
low	27 (40.91)	39 (59.09)		
normal	178 (40.00)	267 (60.00)		
high	10 (27.78)	26 (72.22)		
TgAb			0.528	0.467
high	38 (36.19)	67 (63.81)		
normal	177 (40.05)	265 (59.95)		
TPOAb			0.473	0.491
high	48 (42.11)	66 (57.89)		
normal	167 (38.57)	266 (61.43)		
TSH			0.049	0.976
low	6 (37.50)	10 (62.50)		
normal	198 (39.44)	304 (60.56)		
high	11 (37.93)	18 (62.07)		
Aspect ratio			35.251	<0.001
≤1	148 (33.33)	296 (66.67)		
>1	67 (65.05)	36 (34.95)		
Solid			1.404	0.236
no	7 (28.00)	18 (72.00)		
yes	208 (39.85)	314 (60.15)		
Low echo			61.17	<0.001
no	53 (21.37)	195 (78.63)		
yes	162 (54.18)	137 (45.82)		
Microcalcifications			24.768	<0.001
no	99 (30.65)	224 (69.35)		
yes	116 (51.79)	108 (48.21)		
Morphology			49.477	<0.001
regular	50 (21.93)	178 (78.07)		
irregular	165 (51.72)	154 (48.28)		
Nodule location			3.676	0.159
left	91 (35.97)	162 (64.03)		
right	113 (41.24)	161 (58.76)		
isthmus	11 (55.00)	9 (45.00)		

Reference value of thyroid function: TSH (reference: 0.35–5.00 mIU/ml), TgAb (reference: 0.00–115.00 IU/ml), TPOAb (reference: 0.00–34.00 IU/ml), Tg (reference: 1.40–78.00 mg/L).

### Univariate logistic regression analysis

**Table 1** displays the correlations between medical information and positive FNAB. There were significant differences in age (≤45 years old, P<0.05), nodule size (<1.0 cm, P<0.05), internal or surrounding blood flow signal (yes, P<0.05), aspect ratio (>1, P<0.05), low echo (yes, present, P<0.05), microcalcifications (yes, P<0.05) and morphology (irregular, P<0.05) between the positive-FNAB group and the negative-FNAB group. At the same time, gender, Tg, TgAb, TPOAb, TSH, solid and nodule location were not significantly different between the 2 groups (P>0.05).

### Multivariate logistic regression analysis

After the univariate logistic regression analysis, the above predictors screened out were used for a multivariate logistic regression analysis. As a result, **Table 2** demonstrates that the subsequent variables were found to have independent correlations with a favorable outcome of FNAB: age (≤45 years old) (OR=0.444, P<0.001), low echo (OR=3.549, P<0.001), microcalcifications (OR=2.531, P<0.001), aspect ratio (>1) (OR=3.032, P<0.001) and irregular morphology (OR=2.437, P<0.001).

**Table 2:** Multivariate logistic regression analysis of the relationship between predictors of C-TIRADS 4 thyroid nodules (significant by univariate analysis) and positive FNAB.

Predictors	OR	95% CI	P
Age (year)			<0.001
≤45	Reference	Reference	
>45	0.444	0.296-0.666	
Nodule size (cm)			0.561
<1.0	Reference	Reference	
≥1.0	0.88	0.572-1.354	
Internal or surrounding blood flow signal			0.793
no	Reference	Reference	
yes	0.943	0.609-1.461	
Aspect ratio			<0.001
≤1	Reference	Reference	
>1	3.032	1.819-5.052	
Low echo			<0.001
no	Reference	Reference	
yes	3.549	2.319-5.432	
Microcalcifications			<0.001
no	Reference	Reference	
yes	2.531	1.661-3.856	
Morphology			<0.001
regular	Reference	Reference	
irregular	2.437	1.586-3.745	

### Development of an individualized prediction nomogram

**Figure 5:**
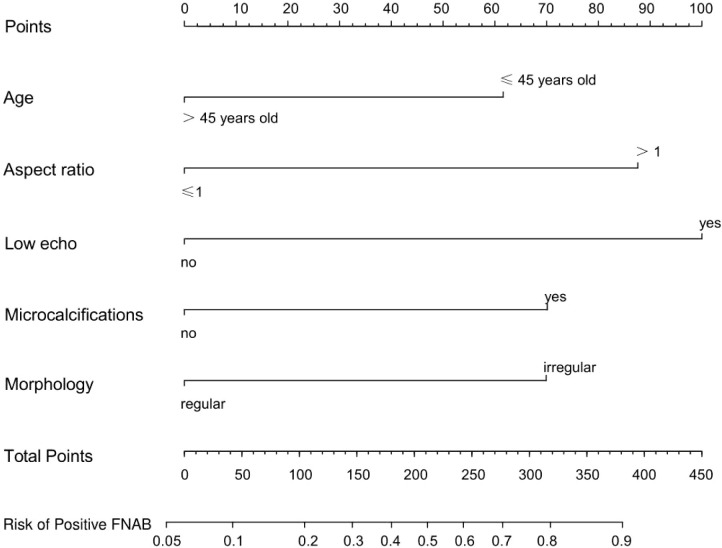
A prediction nomogram. The nomogram is used for the prediction of positive FNAB in C-TIRADS 4 thyroid nodules patients. The prediction nomogram was developed in the cohort, with age, aspect ratio, low echo, microcalcifications and morphology.

The results of the univariate and multivariate logistic regression analyses among age (≤45 years old), low echo, microcalcifications, aspect ratio (>1) and morphology (irregular) are shown in **Table 2**. A nomogram (**Figure 5**) was developed based on the prediction model incorporating the 5 independent predictors mentioned above. **Figure 5** shows that age (≤45 years old) was assigned 62 points, whereas age (>45 years old) received 0 points. A nodal aspect ratio (>1) was assigned 88 points, but a nodal aspect ratio (≤1) received 0 points. A nodule with low echo was assigned 100 points, but a nodule without low echo received 0 points. A nodule with microcalcification was assigned 70 points, and a nodule without microcalcification received 0 points. A nodule with irregular morphology was assigned 70 points, but a nodule with regular morphology received 0 points. The nomogram assigns a maximum of 390 points, and the predictive ability for positive FNAB risk ranges from about 0.05 to 0.9.

### Validation of the prediction nomogram

**Figure 6:**
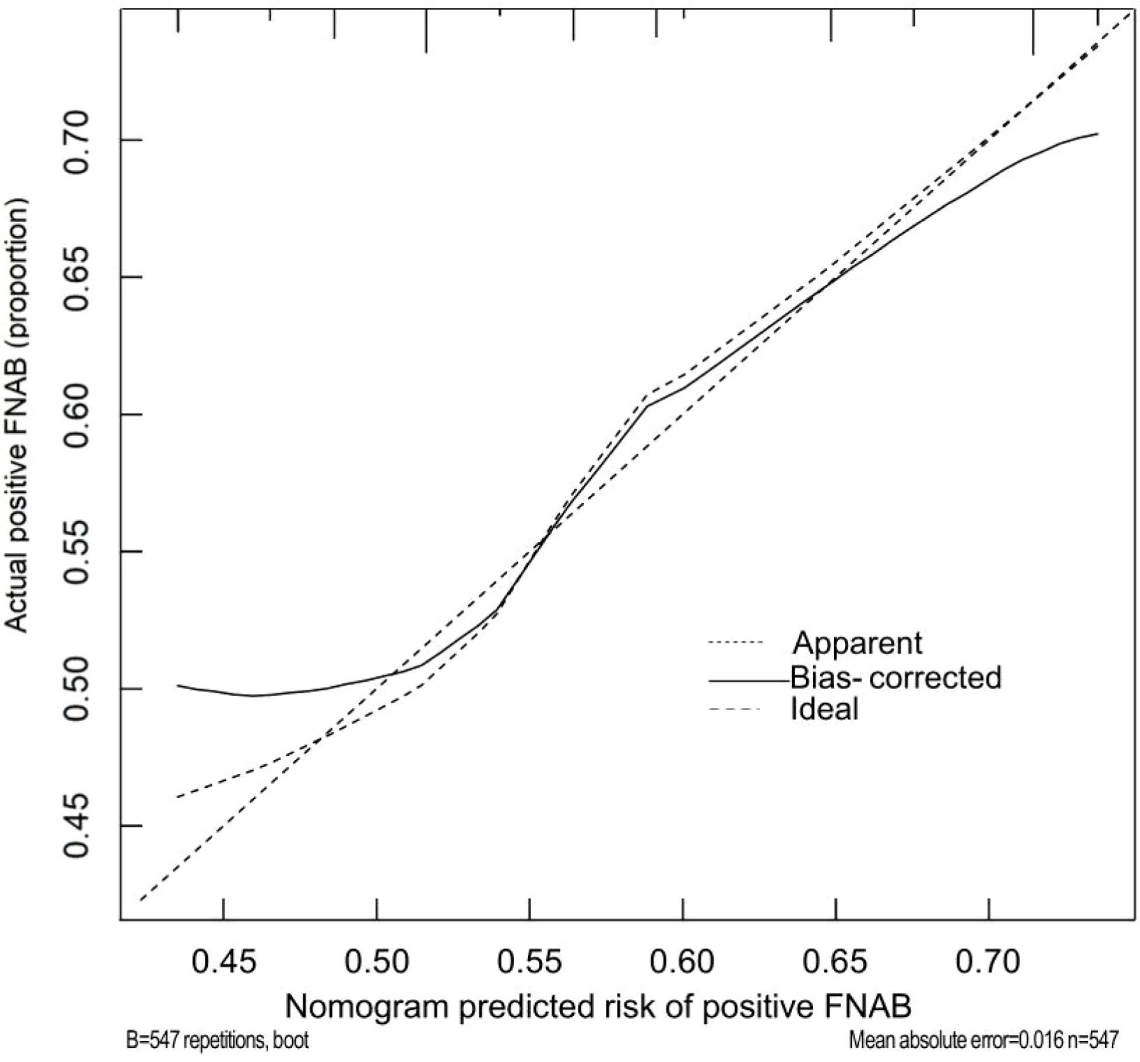
Calibration curves of the prediction nomogram. The solid line is close to the diagonal dotted line, which represents a good prediction ability. The x-axis represents the predicted positive FNAB. The y-axis represents the actual positive FNAB. The diagonal dotted line represents the perfect prediction by the ideal model. The solid line represents the performance of the nomogram, and a closer fit to the diagonal dotted line means a better prediction.

**Figure 7:**
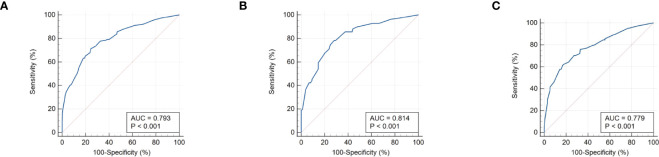
**(A)** The receiver operating characteristics (ROC) curve and area under the curve (AUC) in the training cohort. **(B)** ROC curve and AUC in the validating 1 cohort. **(C)** ROC curve and AUC in the validating 2 cohort. Validating 1 and validating 2 were performed to evaluate the accuracy of this model.

The calibration curve (**Figure 6**) of the nomogram employed for predicting the risk of positive FNAB among patients with C-TIRADS 4 thyroid nodules exhibited satisfactory consistency. The AUC was determined to be 0.793, and it was subsequently validated to be 0.814 and 0.779 through the 2 internal validations (**Figure 7**), which confirmed the model’s good discrimination.

### Clinical use

**Figure 8:**
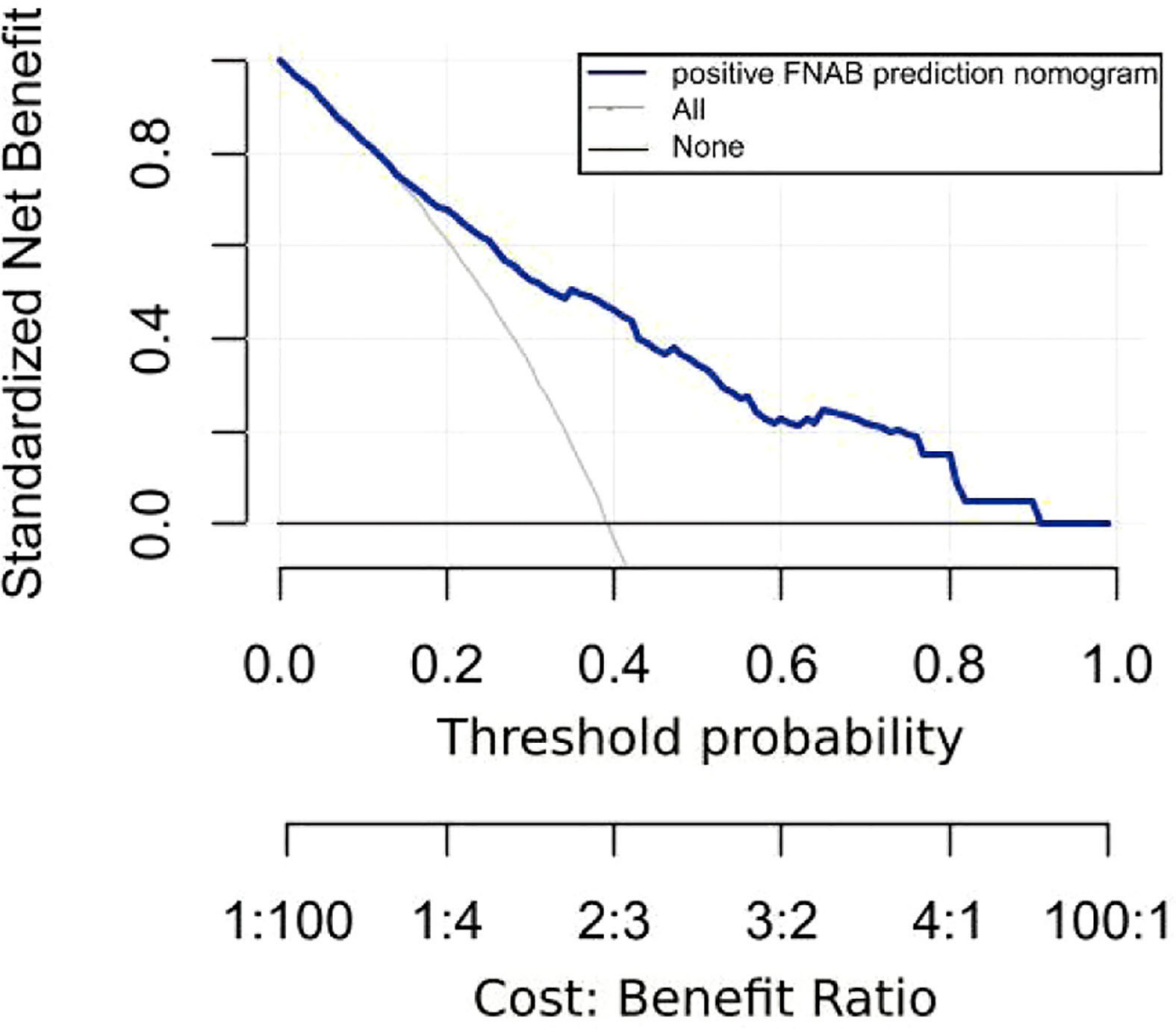
Decision curve analysis (DCA) for the risk nomogram. DCA shows that the model is clinically useful when intervention is decided in the threshold range of 14% to 91%.

**Figure 8** displays the decision curve analysis (DCA) for the risk nomogram. DCA revealed that using the nomogram to predict positive FNAB would be advantageous when the threshold probability ranges between 14% and 91%. In this range, the net benefit was comparable with several overlaps according to the risk nomogram.

## Discussion

Due to the increased use of color Doppler ultrasound, the morbidity of thyroid nodules has increased rapidly in recent decades, even up to 65% in the general population (28). In China, C-TIRADS 4 nodules are mainly considered to be further differentially diagnosed. In this study, 39.3% of the patients’ FNABs were positive, and 60.7% were negative. Similarly, a study showed that at least half of biopsied thyroid nodules are benign (29, 30), and they could be safely managed with annual follow-up (31). TI-RADS is the most effective noninvasive method for evaluating thyroid nodules, and FNAB is currently the most accurate invasive method before surgery (15, 22, 32). In fact, TI-RADS only gives a rough malignant risk (9, 10), and it is related to the ability of ultrasound doctors, which limits its application, especially in primary medical institutions. Studies have shown that ultrasound features, such as low echo, aspect ratio, irregular margins, microcalcifications, and extrathyroidal extension, are related to malignancy (31, 33). In addition, other studies have shown that thyroid function (34, 35), nodule size (15), and age (11, 12) are significantly different between malignant and benign nodules. Therefore, screening out reliable predictors from various medical information and developing a prediction model is vital for C-TIRADS 4 thyroid nodule FNAB, which can reduce at least half of invasive FNAB.

This study included 10 clinical and ultrasound characteristics (age, sex, nodule size, internal or surrounding blood flow signal, aspect ratio, solid, low echo, microcalcifications, morphology and nodule location) and 4 hematological indices (TSH, Tg, TgAb, and TPOAb) as potential predictors for positive FNAB in C-TIRADS 4 thyroid nodules. Previous studies reported microcalcifications, marked low echo, aspect ratio >1, age, sex, nodule size, thyroid function and nodule location as risk factors for positive FNAB (6, 7, 11–14, 21, 36). In this study, age, nodule size, internal or surrounding blood flow signal, aspect ratio, low echo, microcalcifications and morphology were associated with positive FNAB in C-TIRADS 4 thyroid nodules according to the univariate analysis. Additionally, they were not all confirmed as independent predictors through multivariate logistic analysis. Finally, age (≤45 years old), microcalcifications, low echo, aspect ratio (>1) and irregular morphology were confirmed as independent predictors for positive FNAB.

Previous studies have shown that age is associated with positive FNAB (11, 12), which was also confirmed in this study. Norra Kwong et al. reported that the incidence of thyroid carcinoma was 22.9% in the youngest cohort and 12.6% in the oldest cohort (11). Therefore, we believe that age ≤45 years is a risk factor for positive FNAB.

Ultrasound features have a close relationship with thyroid malignancy (37, 38), and this study showed that some ultrasound features, including low echo, aspect ratio >1, microcalcifications and irregular morphology, are independent predictors of positive FNAB.

The echogenicity level of solid nodules includes hyperechoic, isoechoic and hypoechoic. Hypoechoic refers to a thyroid nodule having lower echogenicity compared to the tissue surrounding thyroid (39). Studies have shown that most malignant thyroid nodules are hypoechoic, and the morbidity of malignancy in hypoechoic nodules is higher than that in isoechoic or hyperechoic nodules (40–44). Therefore, low echo is confirmed as an independent predictor in this study.

The aspect ratio is a vital ultrasound feature in determining thyroid nodules. An aspect ratio >1 implies a high possibility of malignancy. In clinical practice, we also find that an aspect ratio >1 takes up a large weight in determining thyroid nodules. This study also confirmed that an aspect ratio >1 is crucial to positive FNAB. In addition, we also concluded that an aspect ratio >1 takes up a large weight from the nomogram.

Microcalcifications are dense calcifications with a single diameter less than 1 mm (39). Under a microscope, thyroid microcalcifications appear as psammoma bodies and they are round, laminar crystalline calcific deposits which are 10-100 μm in size (45). Thyroid microcalcifications are among the most distinctive characteristics of thyroid cancer. Their occurrence is described in follicular and anaplastic thyroid carcinomas (46). In this study, microcalcifications were also included as an independent predictor to construct the nomogram.

Irregular morphology in thyroid nodules refers to characteristics such as irregular or ill-defined margins, where it is challenging to distinguish the boundary between the nodule and surrounding thyroid tissue. It can also include extrathyroidal extension, where the nodule spreads beyond the thyroid capsule, even invading the adjacent soft tissue and/or vascular structures) (39). When a thyroid nodule has a lobulated or irregular margin, its edge appears spiculated or jagged, with or without protrusions into the surrounding parenchyma (47). Irregular morphology is a common manifestation of malignancy. Irregular morphology was also included as an independent predictor of positive FNAB in this study.

In conclusion, we have developed and validated a novel prediction nomogram that utilizes the 5 independent predictors mentioned above. This nomogram allows for personalized prediction of positive FNAB in C-TIRADS 4 thyroid nodules, which can help clinicians in accurately diagnosing malignant thyroid nodules for optimal patient outcomes. The nomogram has been shown to have good discrimination and calibration through 2 internal validations in our cohort. According to DCA, using this nomogram to predict positive FNAB would be beneficial if the threshold probabilities ranged from 14% to 91%. The nomogram is user-friendly and easy to apply clinically for individualized risk assessment. For example, in **Figure 4A**, a 32-year-old patient with a low echo, aspect ratio >1, and irregular morphology had a total score of 320 points, corresponding to an estimated risk of positive FNAB of approximately 81%, indicating a relatively high risk, and FNAB was recommended. Conversely, in **Figure 2B**, a 40-year-old patient with irregular morphology had a total score of 132 points, corresponding to an estimated risk of positive FNAB of approximately 28%, and thus the decision of FNAB should be made more carefully. Finally, FNAB cytological examination indicated inflammatory cells and follicular epithelial cells.

There are certain limitations to this study that should be noted. First, it is a retrospective study, and selection bias is inevitable. Second, the sample size in our study was relatively small. In the future, we will accumulate more cases and detect more indicators to perform more validation and perfection. Third, the ultrasound results and C-TIRADS are largely dependent on the ultrasonic equipment and the examiners’ experience. Fourth, cytological examination also relies on the experience of pathologists. Therefore, subjective factors may lead to more or less bias.

In the study, the factors associated with positive FNAB were analyzed and a predictive model was constructed. However, there was some inconsistency between the positive results of FNAB and the final pathological results. Although the object of this study was positive biopsy, because of the high accuracy of FNAB, it can also be beneficial to the population of thyroid cancer confirmed by postoperative pathology, which can be used as the basis for future research on factors related to thyroid cancer.

## Conclusion

In conclusion, this study has identified 5 independent predictors that are associated with positive FNAB results in C-TIRADS 4 thyroid nodules, which include: age (≤45 years old), low echo, microcalcifications, aspect ratio (>1), and irregular morphology. Additionally, a nomogram was developed based on these predictors, providing clinicians with a tool for quantifying risk assessment and predicting the status of C-TIRADS 4 thyroid nodules before FNAB. This can help to avoid unnecessary treatments and provide a new management strategy for C-TIRADS 4 thyroid nodules.

## Data availability statement

The raw data supporting the conclusions of this article will be made available by the authors, without undue reservation.

## Ethics statement

Ethical review and approval was not required for the study on human participants in accordance with the local legislation and institutional requirements. Written informed consent from the patients was not required to participate in this study in accordance with the national legislation and the institutional requirements.

## Author contributions

LY and ZY were responsible for the study design and preparation of the manuscript. ZY and XG provided the study materials of patients and analyzed all data. LY was responsible for manuscript revisions. All authors have contributed to this article and have given their approval for the final version to be submitted.
